# Impact of Weather and Holidays on Orthopedic Emergency Room Crowding, Fractures, and Polytraumas in a Third-Level Referral Trauma Center in Europe

**DOI:** 10.1155/aort/2970626

**Published:** 2025-01-20

**Authors:** Lorenzo Andreani, Edoardo Ipponi, Francesco Pecchia, Giorgio Balestrieri, Edoardo Tosi, Stefano Marchetti, Paolo Domenico Parchi

**Affiliations:** Department of Orthopedics and Trauma Surgery, University of Pisa, Pisa, Italy

## Abstract

**Background:** Orthopedic trauma is a significant component of emergency department workloads worldwide. The relationship between weather conditions and injury rates is controversial in modern literature. Even less has been written to investigate bank holidays' influence on contusions, dislocations, fractures, and even polytrauma. Our study aimed to assess whether meteorological factors and national holidays could vary the workloads in the orthopedic ER of a European third-level trauma center.

**Materials and Methods:** Our study consisted of a review of all the patients who underwent orthopedic evaluations in our institution's orthopedic emergency room between 2019 and 2023. Days were divided depending on weather (cloudy or sunny vs. rainy or stormy), day type (regular working days vs. national public holidays vs. Saturdays and Sundays), and presence or absence of COVID-19 restrictions. We also recorded the temperatures of each day. Cases were subdivided into three groups: cases without significant injuries (Group A), cases with isolated bone fractures, major tears or articular dislocations (Group B1), and polytrauma (Group B2).

**Results:** Higher temperatures were associated with a significant increase in overall ER visits, isolated injuries, and polytrauma. Sunny or cloudy days had a significantly higher number of patients with all injury types compared to rainy or stormy days. Weekends saw a significant decrease in overall admissions and isolated injuries but a higher rate of polytrauma compared to weekdays. National holidays had a significantly lower number of admissions for all injury types compared to weekdays. Restrictions due to the pandemic significantly reduced overall ER visits.

**Conclusion:** Temperatures, meteorological factors, and national holidays could vary the workloads in the orthopedic ER of a European third-level trauma center.

## 1. Introduction

Orthopedic trauma, encompassing injuries to bones, joints, ligaments, tendons, and muscles, is a significant component of emergency department (ED) caseloads worldwide [1]. In the United States alone, fractures have an incidence that exceeds 2200 per 100,000 person-years and contributes to millions of ED visits every year [2]. These injuries affect all age groups but exhibit distinct patterns based on demographic factors such as age, gender, and activity levels. Children and adolescents are particularly susceptible to fractures and sprains due to high physical activity and sports participation [3]. Sports-related injuries, mainly represented by contusions and sprains, are common among the younger segments of the population and the increasing share of middle-aged people who perform sports regularly [4, 5]. Conversely, older adults face a heightened risk of fractures in the spine and limbs due to osteoporosis and neurological decay, which increases fall propensity [6, 7].

Certain traumas may affect more than one body segment, involving multiple areas and posing a risk to the overall health of the patient; these are known as polytraumas. Polytraumas usually result from high-energy incidents, including motor vehicle accidents, falls from considerable heights, or industrial accidents [8, 9]. Although polytrauma cases represent a smaller percentage of ED visits, they require substantial resources and multidisciplinary care, making them a critical interest for trauma centers [10, 11].

Despite the intuitive influence of meteorological factors such as temperature, rain, or snow on daily living activities, the relationship between weather conditions and injury rates is controversial in modern literature. Even less has been written to investigate bank holidays' influence on contusions, articular distortions, dislocations, fractures, and even polytrauma [12].

Our study aimed to assess whether meteorological factors and national holidays could vary the workloads in the orthopedic ER of a European third-level trauma center.

## 2. Materials and Methods

This single-center retrospective study was performed by the ethical standards laid down in the 1964 Declaration of Helsinki and its later amendments.

Our hospital is a European third-level trauma center with a total capacity of 1080 beds. Located in central Italy, our hospital is the direct referral center for its city and province, which have a population of 98.000 and 421.000 people, respectively. Furthermore, our center is the trauma hub that gathers complex polytraumas from three nearby provinces (890.000 inhabitants in total) located in the same region. Our study consisted of reviewing all the patients who underwent orthopedic evaluations in our institution's emergency room (ER) between January 1, 2019, and March 31, 2023. All cases received a clinical evaluation by an orthopedics and trauma surgeon and eventual X-rays and CT scans to diagnose bone fractures and/or locations. In contrast, ultrasounds were eventually used in case of clinical suspicion of acute tendon or capsule–ligamentous lesions.

Cases were further subdivided into three groups. Group A included those not diagnosed with bone fractures, dislocations, or complete ligament or tendon tears. Group B included those who have been diagnosed with bone fractures or articular dislocations. Those who did not suffer from polytraumas were included in the Sub-Group B1. The Sub-Group B2 included polytraumas, defined as severely injured patients with two or more significant traumatic injuries with a total injury severity score of greater than 15.

For each day in the evaluation interval, we recorded the number of cases evaluated in our orthopedic ER to be included in Group A, Group B1, and Group B2.

We divided days as follows: regular working days (RWDs) (from Monday to Friday), Saturdays and Sundays (SSs) (excluding national holidays), and national public holidays (NPHs). We also considered eventual COVID-19 restrictions, including social distancing and limitations to people transit within the city, region, or country, people like during the evaluated days, which were in force in our city between March 5, 2020, and May 18, 2020, and later between November 7, 2020, and June 21, 2021.

Weather conditions were also retrospectively assessed. The public database on the website “https://www.ilmeteo.it/” enabled us to track the weather in our city on a daily basis. Days were divided into (a) sunny or cloudy days, (b) rainy or heavy rain days, and (c) snowy days.

The high, low, and average temperatures for our hospital's city were recorded in degrees Celsius.

### 2.1. Statistics

We assessed if in our population there was a correlation between weather conditions and/or holidays and the number of total orthopedic ER admissions, bone fractures or articular dislocation, and polytraumas. Statistical analysis was performed using Stata SE 13 (Stata Corp LLC). Statistical significance was set at 0.05 for all endpoints.

## 3. Results

**Figure 1:**
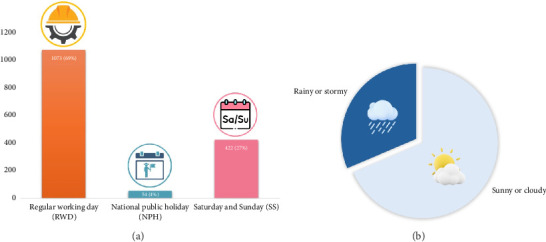
On the left (a) a graphical representation of examined days, divided into regular working days, national public holidays, and Saturdays or Sundays. On the right (b) a summarization of weather conditions, dividing sunny or cloudy days from rainy or stormy days.

Our study evaluated a total of 1549 days. Fifty-four of these days were NPHs, 422 were SSs excluding NPHs, and the remaining 1073 were RWDs (Figure 1(a)).

Out of the 1549 days under scrutiny, 1063 had sunny or cloudy weather, devoid of rainfalls. The remaining 486 days were rainy or stormy (Figure 1(b)).

**Figure 2:**
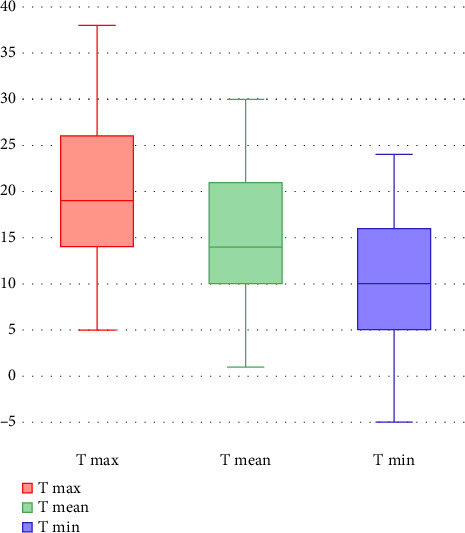
A box plot graph that represents the distributions of higher temperatures (max T, in red), mean temperatures (mean T, in green), and lower temperatures (min T, in blue).

The average mean temperature for the examined days was 15.2°C (1–30). The average lower and higher temperatures were 10.4 (−5–24) and 20.0 (5–38), respectively (Figure 2).

In the examined time interval, 87,637 visits have been performed in our orthopedic ER, with an average attendance of 56.57 (6–110) patients daily. Sixty-eight thousand twenty-five were not diagnosed with fractures, dislocations, or complete tendon and ligament tears (Group 1). A diagnosis of at least one of these conditions, without meeting the criteria for polytrauma (Group 2A), was noted in 17,504 cases. An average of 11.30 (0–27) isolated fractures and comparable lesions were recorded daily in our population.

The number of polytrauma (Group 2B) admitted to our ER during the investigated time was 2108; 1.36 (0–9) per day.

**Figure 3:**
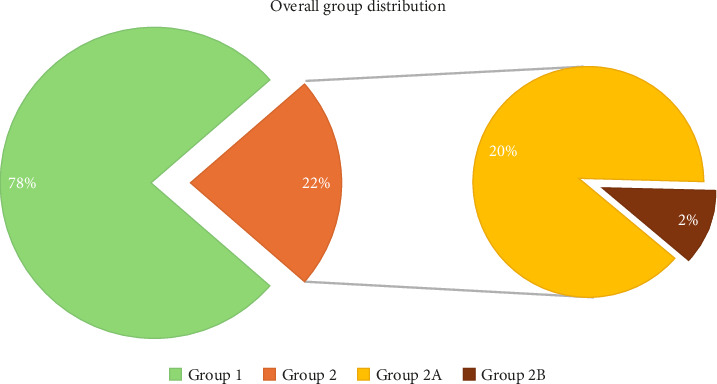
A pie-chart that shows the internal distribution of all the patients evaluated and treated in our orthopedic ER. Group 1 (no diagnosis of fracture, dislocation, or other major lesions) in green and Group 2 (diagnosis of fracture, dislocation, or other major lesions) in orange. Group 2 was further divided into Group 2A (isolated lesions, no polytrauma) and Group 2B (polytrauma).

The distribution of all patients in our groups is resumed in Figure 3.

## 4. The Impact of Temperatures

**Figure 4:**
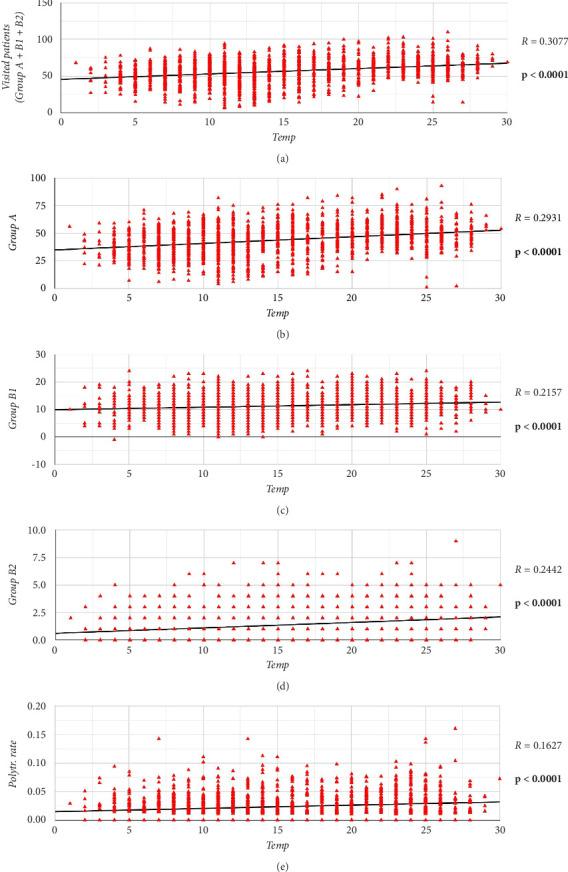
A graphical representation of the linear correlations between mean temperatures (temp) and (a) the number of visited patients, (b) patients belonging to Group A, (c) patients belonging to Group B1, (d) patients belonging to Group B2, and (e) the rate of polytraumas among all treated patients. On the right, the statistical results of each Pearson correlation test. All the reported correlations were statistically significant for the given endpoint (*p* < 0.05).

A Pearson correlation test revealed a direct linear correlation between the mean daily temperature and the number of orthopedic ER visits (*r* = 0.3077; *p* < 0.0001) (Figure 4(a)). Other Pearson correlation tests further indicate that the increase in mean temperatures is also linked to a higher incidence of isolated fractures and major injuries (Group 2A; *r* = 0.2157; *p* < 0.0001) (Figure 4(c)) and polytraumas (Group 2B; *r* = 0.2442; *p* < 0.0001) (Figure 4(d)).

Although our data did not report a significant linear correlation between the rate of isolated fractures (Group 2A) among visited patients and mean temperatures (*r* = 0.0281; *p*=0.2744), these latter were correlated with the rate of polytrauma (Group 2B). A Pearson correlation test also discovered a correlation between the increase in mean temperatures and a higher rate of polytraumas among all the visited patients (*r* = 0.1627; *p* < 0.0001) (Figure 4(e)).

The maximum temperature of each day had a statistically significant direct linear correlation with the number of patients who visited our orthopedic ER, according to a Pearson correlation test: With increased maximum temperatures, more patients attended our orthopedic ER (*r* = 0.3293; *p* < 0.0001). We also detected a similar linear correlation between the lower temperatures and the number of visits performed on each day (*r* = 0.2679; *p* < 0.0001).

A resume of reported statistics is provided in Table 1.

### 4.1. The Impact of Weather Conditions

**Figure 5:**
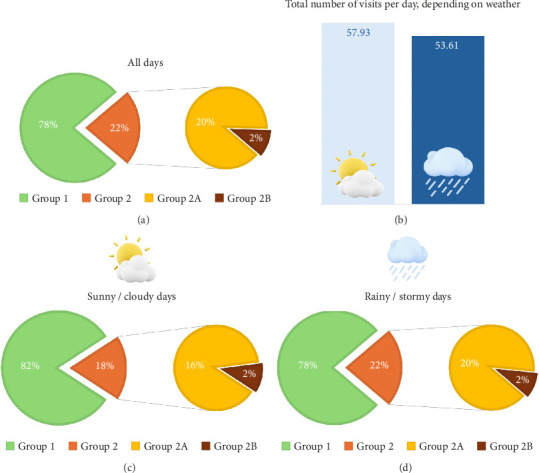
The distribution of visited patients in the various groups among the whole population (a) was graphically compared to the ones of sunny/cloudy days (c) and rainy/stormy days (d). The total number of visits performed during sunny/cloudy ((b), on the left in light blue) and during rainy/stormy days ((b), on the right in dark blue) was also reported graphically.

On sunny and cloudy days without rain, the mean number of patients visited was 57.93 (6–110). On average, 44.96 (6–100) belonged to Group 1, 11.52 (0–24) to Group 2A, and 1.45 (0–9) to Group 2B. On rainy and stormy days, the number of patients visited decreased to 53.61 (14–100) per day. Among them, 41.63 (7–85) belonged to Group 1, 10.82 (0–23) to Group 2A, and 1.15 (0–7) to Group 2B (Figure 5).

In our population, sunny and cloudy days were associated with a significantly higher number of patients admitted to our orthopedic ER compared to rainy days, according to a one-tailed two-sample Mann–Whitney *U* test (*Z* = 5.325; *p* < 0.0001). The more significant number of admitted patients also translated into a significantly higher daily incidence of localized fractures, dislocations, or major tears (one-tailed Student's t-test *T* = 4.1562; *p* < 0.0001) but also polytraumas (one-tailed two-sample Mann–Whitney *U* test *Z* = 4.2384; *p* < 0.0001) during sunny and cloudy days compared to rainy and stormy days. The ratio of Group 2A among all visited patients did not change depending on the weather (two-tailed two-sample Mann–Whitney *U* test *Z* = 0.1039; *p*=0.9172). Conversely, a one-tailed two-sample Mann–Whitney *U* test reported that sunny and cloudy days had significantly higher rates of polytraumas among all visits (*Z* = 3.3380; *p*=0.0004). A summary of these results is portrayed in Table 2.

## 5. The Impact of Weekends and Holidays

**Figure 6:**
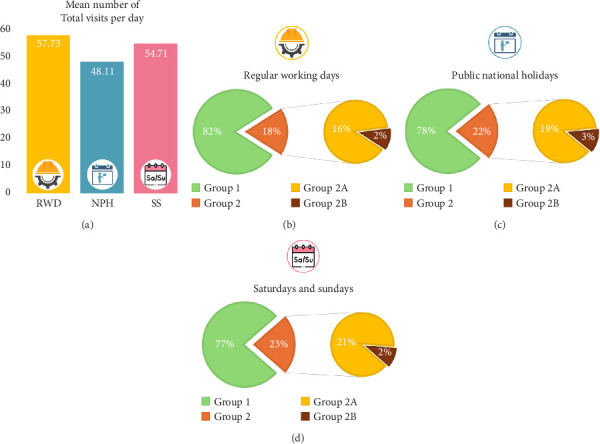
On top (a), a graphic representation of the mean number of visits performed in our orthopedic ER during regular working days (RWDs), national public holidays (NPWs), and Saturdays or Sundays (SSs). Below, pie-charts to describe the internal distribution of all the patients evaluated and treated (green = Group 1, orange = Group 2, yellow = Group 2A, and brown = Group 2B) during RWDs (b), PNHs (c), and SSs (d).

The mean number of patients visited in our orthopedic ER on RWDs (Monday through Friday, excluding national holidays) was 57.73 (8–110). The average number of patients belonging to Group 1, Group 2A, and Group 2B was 44.64 (1–93), 11.76 (0–24), and 1.33 (0–8), respectively (Figures 6(a) and 6(b)).

On regular weekend days (SSs excluding national holidays), the mean number of patients visited in our orthopedic ER was 54.71 (6–103). The average number of patients belonging to Group 1, Group 2A, and Group 2B was 42.86 (1–93), 10.39 (0–23), and 1.45 (0–9), respectively (Figures 6(a) and 6(d)).

On NPHs, the mean number of patients visited in our orthopedic ER was 48.11 (15–94) patients per day. On average, 37.65 (11–82) of them belonged to Group 1, 9.15 (2–18) to Group 2A, and 1.31 (0–7) to Group 2B (Figures 6(a) and 6(c)).

According to one-tailed two-sample Mann–Whitney *U* tests, the mean total number of visited patients was significantly higher on RWDs compared to SSs or NPHs (*Z* = 3.3728; p = 0.0003 and *Z* = 4.1485; *p* < 0.0001, respectively).

The same tests reported a significantly higher incidence of isolated lesions (Group 2A) on RWDs compared to SSs or NPHs (*Z* = 4.7077; *p* < 0.0001 and *Z* = 4.5410; *p* < 0.0001, respectively). A two-tailed Mann–Whitney *U* test confirmed that the diagnosis of isolated lesions was significantly more common during RWDs than during SSs (*Z* = 3.2203; *p*=0.0006). A similar test did not highlight a significantly decreased relative risk of isolated lesions on NPHs compared to RWDs (*Z* = 0.0184; *p*=0.4926).

According to a two-tailed Mann–Whitney *U* test, the difference in the daily incidence of polytraumas during RWDs and NPHs was not statistically significant (*Z* = 1.01; *p*=0.1562).

The mean daily polytrauma (Group 2B) was significantly higher on SSs than on RWDs (one-tailed Mann–Whitney *U* test; *Z* = 1.8754; *p*=0.0304). Another one-tailed Mann–Whitney *U* test evidenced that the daily rate of polytraumas was significantly higher on SSs compared to RWDs (*Z* = 2.7595; *p*=0.0029).

A summary of these results is reported in Table 3.

### 5.1. The Impact of COVID-19

**Figure 7:**
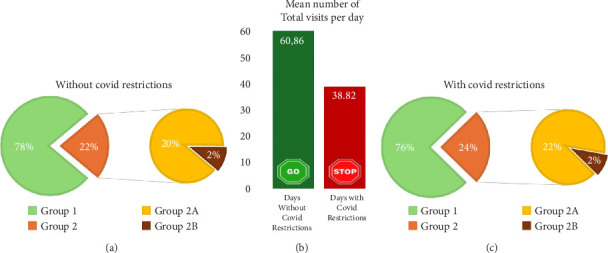
On the left (a), and on the right (c) pie-charts to describe the internal distribution of all the patients evaluated and treated (green = Group 1, orange = Group 2, yellow = Group 2A, and brown = Group 2B) during days respectively without and with COVID-19 restrictions. In the middle (b) a graphic representation of the mean number of visits performed in our orthopedic ER during days without restrictions (green column) and days with restrictions due to the COVID-19 epidemics (red column).

Among the 1549 days evaluated in our study, 301 were conditioned by restrictions caused by the global COVID-19 epidemic and consequential limitations in people's activities, distancing, and travel. During these days, the mean number of visited patients was 38.82 (6–81), whereas the mean number of visits in the remaining days was 60.86 (14–110). This difference was statistically significant according to a one-tailed, two-sample Mann–Whitney *U* test (*Z* = 19.4752; *p* < 0.0001). The distribution of patients on days with COVID-19 restrictions was as follows: Group A had an average of 29.37 patients (4–62), Group B1 had 8.56 (0–24), and Group B2 had 0.89 (0–6). A graphical comparison of the distribution between the three groups on days with and without COVID-19 restrictions is reported in Figure 7.

In our population, the rate of patients with isolated fractures or other major musculoskeletal lesions (Group 2A) among all visited patients had significantly increased during the days with COVID-19 restrictions (one-tailed Mann–Whitney *U* test; *Z* = 4.3578; *p* < 0.0001). Conversely, the daily rate of polytrauma (Group 2B) among all visited patients was significantly lower during the days with COVID-19 restrictions than on the other days (one-tailed Mann–Whitney *U* test; *Z* = 2.8192; *p*=0.0024).

A summary of these statistical findings is provided in Table 4.

## 6. Discussion

Since the dawn of civilization, weather has always influenced human activities [13–15]. Climatic conditions imply variations in activities, such as working, driving, and performing sports that represent some of the most common settings for musculoskeletal injuries [14–17]. Consequently, climate and temperatures could directly or indirectly influence the incidence of traumas, thereby increasing or reducing the workload of trauma centers and orthopedics ERs.

In 2015, Ali and Willett [12] conducted a systematic review examining the impact of weather on trauma workload. Their findings provided strong evidence that higher temperatures are positively associated with an increase in trauma admissions. Atherton et al. [18], Bhattacharya et al. [19], Friede et al. [20], Parsons et al. [21], and Rising et al. [22] all discovered a correlation between temperatures and trauma admissions over populations of 8000 or more cases. All these authors indicated the maximum daily temperature as the most important predictor for the local incidence of musculoskeletal injuries that required visits and hospitalization. Our experience aligns with the ones reported in previous literature, as we found a statistically significant linear correlation between maximum temperatures in a day and the number of visits performed in our orthopedic ER (*r* = 0.3293; *p* < 0.0001). Similar evidence, although with a slightly lower correlation level, linked the number of all admissions with the minimum temperatures (*r* = 0.2679; *p* < 0.0001) as well as the mean daily temperatures (*r* = 0.3077; *p* < 0.0001). These robust findings suggest that the rise in temperatures may directly impact the workload of orthopedic ERs, especially if located in regions that are not predominantly mountainous and without large-scale ski areas and winter sports facilities. Focusing on mean temperatures, we also discovered that temperature increment also had linear correlations with the rates of both localized musculoskeletal injuries and polytrauma (*p* < 0.0001). Hence, higher temperatures not only lead to more admissions, but among these cases, it increases the share of localized fractures, dislocations, tendon and ligament tears, and polytraumas. This evidence is particularly concerning, also considering that global warming promises to further increase the temperatures in the years to come [23].

Less has been written about the influence of good or bad weather on the workload in trauma centers, as their role is still debated. Bhattacharya et al. [19] and Friede et al. [20] reported that rainfalls were associated with fewer admissions in their populations. Jacobsen et al. [24] and Tenias et al. [25], for their part, did not highlight any significant effect of rain on the daily incidence of hip fractures in a sample of over one thousand patients. Conversely, adverse weather conditions increased adult trauma admissions in the paper by Parsons et al. [21]. Rising et al. [22] also discovered precipitation to be a valid predictor of increased trauma admission volume, with a 60% to 78% increment in the incident rate for each inch of precipitation in the previous 3 h. In our experience, rainy and stormy days significantly reduced global admissions, fractures, and polytraumas (*p* < 0.0001). Furthermore, not only did rains and storms reduce the overall polytraumas, but they also led to a significant reduction in polytrauma rates among all visited patients (*p*=0.0004). These outcomes support the idea that weather conditions impact the workload in trauma centers, whose workload should be expected to increase during sunny and cloudy days.

Precipitations and temperatures are not the only factors that change a population's behavior from one day to another, and not all days are the same.

In modern Western civilization, many working activities occur from Monday to Friday, the so-called “working days.” In contrast, Saturdays and especially Sundays are primarily reserved for personal time rather than work-related activities. Likewise, national holidays are typically viewed as chances to enjoy extra leisure time, often accompanied by various celebrations. The differences in lifestyle between weekdays and weekends could theoretically lead to significant fluctuations in the workload of trauma centers. Only a few studies have investigated the impact of weekends on orthopedic activity in trauma centers. Nijland et al. [26] and Boutera et al. [27] had comparable incidences of hip fractures during weekends and working days, whereas Johansen et al. [28] even described a reduction in the number of hip fracture diagnoses during weekends. Conversely, some authors described an increased incidence of pediatric and sports injuries on weekends compared to working days [29, 30]. In our experience, we observed a significantly lower number of admissions on weekends compared to working days (*p*=0.0003). This correlation was attributable to lower incidences of both cases without relevant injuries and with localized musculoskeletal lesions. Instead, the daily mean number of polytrauma (*p*=0.0304) and the rate of polytrauma among all admissions (*p*=0.0029) were significantly higher on weekends. Our findings suggest that weekend lifestyle and behavior prevent minor injuries but simultaneously increase the chances of high energy and multidistrict trauma in a heterogeneous population.

Like weekends, national holidays are often seen as opportunities to extend leisure time, along with various kinds of celebrations. The link between holidays and the workload in the orthopedic ERs of trauma centers is controversial. On the one hand, some studies documented an increased incidence of trauma during holidays in both the adult and pediatric populations [31, 32]. On the other hand, Abdelrahman et al. [33] did not find significant variations in trauma incidence during holidays. Our data are in contrast with the ones reported in previous literature, testifying that the incidence of admissions in general, isolated musculoskeletal injuries and polytrauma, is significantly lower during NPHs compared to RWDs. According to our findings, bank holidays should not be considered high-risk days, especially for south European and Mediterranean countries.

Finally, our outcomes confirmed the impact that the COVID-19 epidemic had on the activities of trauma centers worldwide. Restrictions imposed to prevent the spread of the disease led to a significant reduction in the overall number of visits performed in our orthopedic ER. This result aligns with most previous studies that reported a reduction in trauma centers, especially during the first year of the epidemic [34–36]. Furthermore, the significant reduction in localized musculoskeletal injuries and polytrauma rates suggests that the decreased daily incidence was mainly attributable to the changes in everyday lifestyle rather than the fear of contagion [36].

We acknowledge that our study has some limitations. Our sources did not allow us to record the millimeters of rainfall, impeding the evaluation of the rain intensity as a possible predictive factor. The same goes for rain duration, which could not be assessed. Another issue was the inability to precisely determine the lag between the onset of the trauma and the admission to our orthopedic ER. Prolonged delays may have caused a slight distortion in our data. Patients who were injured due to adverse weather or activities carried out under specific climatic conditions or scheduled events might have been recorded the following days. Another constraint is the absence of data regarding the traumatic mechanism and its relative frequency depending on temperatures, weather, working days, weekends, or holidays. The inclusion of such data is desirable for the studies to come to allow further confirmations of eventual cause–effect relationships between traumatic events and modifications in the number of admissions, fractures, and polytrauma. One more limitation of our study is its monocentric nature. Involving only one hospital, our investigation is strictly linked to the characteristics of our territory and the cultural traits of its inhabitants. Further multicentric studies, including trauma centers with different landscapes, climatic settings, and cultural contexts, would overcome this issue and increase the overall number of observed patients.

Beyond these limitations, our study provides an unprecedented overview of the impact of climatic conditions, including temperatures and rainfalls, and the distinction between RWDs and weekends or bank holidays on the workload of an orthopedic ER in a third-level trauma center in Europe. We found that higher temperatures and sunny or cloudy days significantly increased minor and major musculoskeletal traumas.

We also proved a significant reduction in the overall workload during holidays and weekends, although these latter were prone to significantly more frequent polytraumas. Our data and outcomes could help national health systems and single institutions predict the number of admissions and critical patients, allocating both human and nonhuman resources accordingly. In a world where finances for trauma activities are often kept to a minimum and temperatures are constantly increasing, examining the changes in the climate and our habits will be essential to provide the best standard of care for our patients.

## Figures and Tables

**Table 1:** A schematic summary of the statistical tests performed to find statistically significant linear correlations between temperatures and visits in our orthopedic ER.

Test	Data 1	Data 2	Stat. results
Pearson correlation test	Mean temperature	All visits	Higher mean temperatures lead to significantly more visits (*r* = 0.3077; **p** < 0.0001)
Pearson correlation test	Mean temperature	All 1s	Higher mean temperatures lead to significantly more 1s (*r* = 0.3077; **p** < 0.0001)
Pearson correlation test	Mean temperature	All 2As	Higher mean temperatures lead to significantly more 2As (*r* = 0.2157; **p** < 0.0001)
Pearson correlation test	Mean temperature	All 2Bs	Higher mean temperatures lead to significantly more 2Bs (*r* = 0.2442; **p** < 0.0001)
Pearson correlation test	Mean temperature	All 2A/all	Higher mean temperatures did not lead to significantly more 2A/all (*r* = 0.0281; *p*=0.2744)
Pearson correlation test	Mean temperature	All 2B/all	Higher mean temperatures did not lead to significantly more 2B/all (*r* = 0.1627; **p** < 0.0001)
Pearson correlation test	Maximum temperature	All visits	Higher max temperatures lead to significantly more visits (*r* = 0.3293; **p** < 0.0001)
Pearson correlation test	Minimum temperature	All visits	Higher min temperatures lead to significantly more visits (*r* = 0.2679; **p** < 0.0001)

*Note:* Significance cutoff set to *p* < 0.05.

**Table 2:** A schematic summary of the statistical tests performed to find statistically significant differences in the number of visits between sunny/cloudy days and rainy/stormy days.

Test	Data 1	Data 2	Stat. results
One-tailed Mann–Whitney *U* test	All visits on sunny and cloudy days	All visits on rainy and stormy days	Significantly more visits on sunny and cloudy days (*Z* = 5.325; **p** < 0.0001)
One tailed T-student test	2A on sunny and cloudy days	2A on rainy and stormy days	Significantly more 2As on sunny and cloudy days (*T* = 4.1562; **p** < 0.0001)
One-tailed Mann–Whitney *U* test	2A/all on sunny and cloudy days	2A/all on rainy and stormy days	No statistically significant difference between 2A/all depending on weather (*Z* = 0.1039; *p*=0.9172)
One-tailed Mann–Whitney *U* test	2B on sunny and cloudy days	2B on rainy and stormy days	Significantly more 2Bs on sunny and cloudy days (*Z* = 4.2384; **p** < 0.0001)
One-tailed Mann–Whitney *U* test	2B/all on sunny and cloudy days	2B/all on rainy and stormy days	Significantly more 2B/all on sunny and cloudy days (*Z* = 3.3380; **p**=0.0004)

*Note:* Significance cutoff set to *p* < 0.05.

**Table 3:** A schematic summary of the statistical tests performed to find statistically significant differences in the number of visits between regular working days (RWDs), Saturdays and Sundays (SSs), and public national holidays (PNHs).

Test	Data 1	Data 2	Stat. results
One-tailed Mann–Whitney *U* test	All visits on RWDs	All visits on SSs	Significantly more visits on RWDs (*Z* = 3.3728; **p**=0.0003)
One-tailed Mann–Whitney *U* test	All visits on RWDs	All visits on PNHs	Significantly more visits on RWDs (*Z* = 4.1485; **p** < 0.0001)
One-tailed Mann–Whitney *U* test	2A on RWDs	2A on SSs	Significantly more 2As on RWDs (*Z* = 4.7077; **p** < 0.0001)
One-tailed Mann–Whitney *U* test	2A on RWDs	2A on or PNHs	Significantly more 2As on RWDs (*Z* = 4.5410; **p** < 0.0001)
Two-tailed Mann–Whitney *U* test	2A/all on RWDs	2A/all on SSs	Significantly more 2A/all on RWDs (*Z* = 3.2203; **p**=0.0006)
Two-tailed Mann–Whitney *U* test	2A/all on RWDs	2A/all on PNHs	No statistically significant difference between 2A/all on RWDs and PNHs (*Z* = 0.0184; *p*=0.4926)
Two-tailed Mann Whitney *U* test	2B on RWDs	2B on PNHs	No statistically significant difference between 2B on RWDs and PNHs (*Z* = 1.01; *p*=0.1562)
One-tailed Mann–Whitney *U* test	2B on RWDs	2B on SSs	Significantly more 2B on SS (*Z* = 1.8754; **p**=0.0304)
One-tailed Mann–Whitney *U* test	2B/all on RWDs	2B/all on SSs	Significantly more 2B/all on SS (*Z* = 2.7595; **p**=0.0029)

*Note:* Significance cutoff set to *p* < 0.05.

**Table 4:** A schematic summary of the statistical tests performed to find statistically significant differences in the number of visits between regular working days (RWDs), Saturdays and Sundays (SSs), and public national holidays (PNHs).

Test	Data 1	Data 2	Stat. results
One-tailed Mann–Whitney *U* test	All visits on days without COVID-19 restrictions	All visits on days with COVID-19 restrictions	Significantly less visits on days without COVID-19 restrictions (*Z* = 19.4752; **p** < 0.0001)
One-tailed Mann–Whitney *U* test	2A/all visits on days without COVID-19 restrictions	2A/all visits on days with COVID-19 restrictions	Significantly more 2A/all visits on days without COVID-19 restrictions (*Z* = 4.3578; **p** < 0.0001)
One-tailed Mann–Whitney *U* test	2B/all visits on days without COVID-19 restrictions	2B/all visits on days with COVID-19 restrictions	Significantly less 2B/all visits on days without COVID-19 restrictions (*Z* = 2.8192; **p**=0.0024)

*Note:* Significance cutoff set to *p* < 0.05.

## Data Availability

The data that support the findings of this study are available from the corresponding author upon reasonable request.
